# Artemisinin-based combination therapy for uncomplicated *Plasmodium falciparum* malaria in Mali: a systematic review and meta-analysis

**DOI:** 10.1186/s12936-021-03890-0

**Published:** 2021-08-30

**Authors:** Fatoumata O. Maiga, Mamadou Wele, Sounkou M. Toure, Makan Keita, Cheick Oumar Tangara, Randi R. Refeld, Oumar Thiero, Kassoum Kayentao, Mahamadou Diakite, Antoine Dara, Jian Li, Mahamoudou Toure, Issaka Sagara, Abdoulaye Djimdé, Frances J. Mather, Seydou O. Doumbia, Jeffrey G. Shaffer

**Affiliations:** 1 University of Sciences, Techniques and Technologies of Bamako, Bamako, Mali; 2 Department of Biostatistics and Data Science, Tulane University School of Public Health and Tropical Medicine, 1440 Canal Street #8310, Suite 1610, New Orleans, LA 70112-2703 USA

**Keywords:** Artemether–lumefantrine, Artemisinin-based combination therapy, Malaria, Mali, Systematic review

## Abstract

**Background:**

Artemisinin-based combination therapy (ACT) was deployed in 2005 as an alternative to chloroquine and is considered the most efficacious treatment currently available for uncomplicated falciparum malaria. While widespread artemisinin resistance has not been reported to date in Africa, recent studies have reported partial resistance in Rwanda. The purpose of this study is to provide a current systematic review and meta-analysis on ACT at Mali study sites, where falciparum malaria is highly endemic.

**Methods:**

A systematic review of the literature maintained in the bibliographic databases accessible through the PubMed, ScienceDirect and Web of Science search engines was performed to identify research studies on ACT occurring at Mali study sites. Selected studies included trials occurring at Mali study sites with reported polymerase chain reaction (PCR)-corrected adequate clinical and parasite response rates (ACPRcs) at 28 days. Data were stratified by treatment arm (artemether–lumefantrine (AL), the first-line treatment for falciparum malaria in Mali and non-AL arms) and analysed using random-effects, meta-analysis approaches.

**Results:**

A total of 11 studies met the inclusion criteria, and a risk of bias assessment carried out by two independent reviewers determined low risk of bias among all assessed criteria. The ACPRc for the first-line AL at Mali sites was 99.0% (95% CI (98.3%, 99.8%)), while the ACPRc among non-AL treatment arms was 98.9% (95% CI (98.3%, 99.5%)). The difference in ACPRcs between non-AL treatment arms and AL treatment arms was not statistically significant (p = .752), suggesting that there are potential treatment alternatives beyond the first-line of AL in Mali.

**Conclusions:**

ACT remains highly efficacious in treating uncomplicated falciparum malaria in Mali. Country-specific meta-analyses on ACT are needed on an ongoing basis for monitoring and evaluating drug efficacy patterns to guide local malaria treatment policies, particularly in the wake of observed artemisinin resistance in Southeast Asia and partial resistance in Rwanda.

## Background

Falciparum malaria is the most deadly type of malaria and causes most cases of malaria in sub-Saharan Africa [1, 2]. For over four decades, chloroquine was the first-line treatment of uncomplicated falciparum malaria, and molecular markers for its resistance were first observed in 2001 [3]. Artemisinin-based combination therapy (ACT) has since replaced chloroquine due to widespread resistance [4, 5]. Between 2001 and 2004, ACT was launched in 20 African countries in response to World Health Organization (WHO) recommendations [6]. ACT is currently used in Africa, Asia and South America [7] and is recommended as first-line treatment of uncomplicated falciparum malaria [8]. Since 2005, 81.5% (25.5 of 31.3 million) of ACT courses deployed globally were distributed in Africa [9]. ACT is deployed as a combination therapy as the use of artemisinin monotherapy has been shown to promote the development of artemisinin resistance [10]. Most ACT failures are a result of re-infections [11], and the most widely used approach for assessing ACT efficacy is the in vivo approach at 28 days following treatment with molecular correction for re-infection [12]. While ACT is intended for malaria treatment, it has also been efficacious in preventing transmission [13].

WHO currently recommends five artemisinin-based combinations: artesunate-amodiaquine (AS + AQ); artesunate–mefloquine (AS + MQ); artesunate–sulfadoxine–pyrimethamine (AS + SP); artemether–lumefantrine (AL); and, dihydroartemisinin–piperaquine (DHA + PQ) [8]. A newer ACT, artesunate–pyronaridine (AS + Pyr) is being considered for use where others are failing [8]. Artesunate–atovaquone–proguanil (AS + AP) is not usually used in endemic areas due to the high cost of atovaquone [14]. Artesunate–chlorproguanil–dapsone (AS + CD) is no longer used in African settings due to its haemolytic potential in glucose-6-phosphate dehydrogenase (G6PD)-deficient patients [15]. Artesunate–sulfamethoxypyrazine–pyrimethamine (AS + SMP) is widely used in Central African markets but is not recommended by the WHO for treating falciparum malaria [16].

### Artemisinin combination therapy in Mali

Malaria remains a substantial burden in Mali and is its leading cause of morbidity and mortality in children aged under 5 years [17, 18]. In 2005, a pilot campaign for free ACT (AS + AQ) was introduced in Mali by Doctors Without Borders (abbreviated as MSF for its French translation: *Médecins Sans Frontières*) [19]. Later in 2005, Mali officially adopted AL ACT as a replacement for chloroquine [20]. AL remains the recommended first-line treatment for uncomplicated falciparum malaria in Mali, with AS + AQ as second-line treatment [20, 21].

### Systematic reviews and meta-analyses as evaluation tools for artemisinin-based combination therapy

Systematic reviews and meta-analyses have routinely been performed over the past decade to monitor and assess ACT efficacy, tolerability and adherence for African regions. In 2004, a meta-analysis of 16 clinical trials evaluated the effects of adding artesunate to standard anti-malarial treatments, such as amodiaquine [22]. In 2009, a review of 50 ACT studies in Asia and Africa revealed that all five ACT in use at the time yielded treatment failure rates of under 10% at most study sites, which met WHO guidelines [23]. Another meta-analysis focusing on sub-Saharan Africa study sites showed that ACT yielded lower failure rates (relative to oral quinine) in second and third-trimester pregnancies [24]. A review of 11 studies on the repeated dosages of DHA + PQ in pregnant women showed the regimen to be well tolerated as an intermittent preventive treatment (IPT) [25]. A larger meta-analysis of 78 studies focusing on drug resistance to falciparum malaria revealed that ACT was less prone to drug resistance than non-ACT (NACT) [26]. Another review of 76 studies conducted in sub-Saharan Africa published between 2002 and 2016 revealed that, among the WHO-recommended ACT, DHA + PQ was the most efficacious [27].

### Recent country-specific meta-analyses in African countries

Country-specific meta-analyses for ACT have recently been performed for several African countries. A review of 13 studies conducted in Uganda between 2002 and 2010 showed that AL was highly efficacious in Uganda, with efficacy rates of 98% [28]. In 2017, a meta-analysis including study sites in Ethiopia showed that anti-malarial treatment success rates were 92.9%, and standard regimens showed high success rates against both *Plasmodium falciparum* (98.1%) and *Plasmodium vivax* (94.7%) infections [29]. In 2018, a meta-analysis showed that ACT remains highly efficacious in Sudan, where overall malaria treatment success rates were 98.0%, and the AL regimen showed higher efficacy compared to AS + SP [30]. In 2019, a network meta-analysis of six studies in Cameroon showed anti-malarial success rates were between 88.2 and 100% [31]. In a recent review of ACT efficacy at Guinea sites, each of the three included studies reported ACT efficacy rates over 95% [32].

Artemisinin resistance has recently been observed in Southeast Asia, where, in 2020, a network meta-analysis of 82 studies reported artemisinin resistance [33]. While widespread artemisinin resistance has not been reported in Africa, at least one case study has reported resistance [34]. A recent review study in Burkina Faso did not reveal ACT resistance, but one of the reviewed studies raised concern about the possibility [35, 36]. Evidence of emerging artemisinin partial resistance has been observed in Rwanda, and the evaluation of additional anti-malarials in Rwanda has been recommended [37]. A recent study has shown evidence for the de novo emergence of *Pfkelch13*-mediated resistance in Rwanda, potentially compromising the continued success of anti-malarial chemotherapy in Africa [38]. Additionally, recent studies in Mali have also shown an increased frequency of recurrent parasitaemia following AL treatment [39]. Recent studies such as these suggest that African countries may be on the verge of meaningful artemisinin resistance, as previously observed in Southeast Asia [40].

While country-specific meta-analyses have recently been carried out for Sudan, Ethiopia, Cameroon, Burkina Faso, and Guinea, an analogue study has yet to be performed for Mali, where falciparum malaria rates are among the most prevalent and burdensome in the world. Recent reports showing the emergence of artemisinin resistance illustrate the need for monitoring and evaluating the efficacy of ACT to guide local health policy and decision making. This systematic review aims to accentuate the current efficacy of ACT in Mali and provide much-needed information on potential artemisinin resistance, which is currently lacking in Mali. The aim of this study, therefore, is to fill this gap through a systematic review and meta-analysis of ACT trials carried out at Mali study locations. More specifically, the study aims to evaluate the current efficacy of first-line ACT in Mali and determine whether other treatments are equally efficacious for alternative candidate therapy. To our knowledge, this is the first systematic review of ACT focused exclusively on Mali study locations.

## Methods

A systematic review and meta-analysis of ACT trials was performed according to the established Preferred Reporting Items for Systematic Reviews and Meta-Analyses (PRISMA) guidelines [41]. The review focused on evaluating the overall efficacy of first-line AL ACT and other ACT deployed at Mali study sites. A systematic review of the literature was performed by querying bibliographic databases indexed by the PubMed, ScienceDirect and Web of Science search engines. These queries were performed without any time or language restrictions. The search terms were input as: (“Mali” and “artemisinin” and ACPR”) or (“Mali” and “artemisinin-based and (“trial” or “randomized”)) or (“Mali” and “artemether” and “lumefantrine” and (“trial” or “randomized”)). “Therapies” was purposely omitted as its plurality varied across studies. The latter search component (“Mali” and “artemether” and “lumefantrine” and (“trial” or “randomized”)) was used because AL is currently the first-line treatment for uncomplicated falciparum malaria in Mali. Further inclusion criteria required at least one ACT comparison arm, the ability to disaggregate study results for Mali locations, and inclusion of the primary outcome (absence of parasitaemia at day 28 irrespective of axillary temperature and without early or late treatment failure or late parasitological failure corrected by PCR (adequate clinical and parasite response (ACPRc)) [42]. Publications on editorials, guidelines and theoretical articles were excluded from the final selected studies.

### Study outcomes and data extraction

The primary outcome was ACPRc at 28 days following treatment. ACPRc data were abstracted by study arm for each of the selected studies. Other data abstracted included author names, year of publication, journal name and type, treatment arm type, treatment regimen, study design type, study location, age range inclusion criteria, analysis type (intention to treat or per protocol), parasitaemia at day 28 irrespective of axillary temperature and without early or late treatment failure or late parasitological failure (uncorrected ACPR, referred to here as ACPR) and their respective confidence intervals, and ACPRcs and their respective confidence intervals. Study locations were geocoded and mapped.

### Meta-statistical analysis

Data were expressed as frequencies, percentages and standard errors (SEs). Outcomes (ACPRs and ACPRcs) were rounded to one decimal place as it was the most consistent reporting method for the primary outcome in the selected studies. Confidence intervals and SEs were calculated according to the normal approximation formula for a single proportion $$\left( {SE = \sqrt {{\raise0.7ex\hbox{${p\left( {1 - p} \right)}$} \!\mathord{\left/ {\vphantom {{p\left( {1 - p} \right)} n}}\right.\kern-\nulldelimiterspace} \!\lower0.7ex\hbox{$n$}}} } \right)$$, where *p* and *n* represent the reported proportions and sample sizes, respectively. Sample sizes were considered as the total number of subjects evaluated (number of enrolled subjects excluding withdrawals) by study arm. Confidence intervals were based on a standard normal distribution with a 5% type I error rate and calculated as 1.96 times the standard error for each reported proportion. Heterogeneity was assessed according to Cochran’s Q and I^2^ tests [43]. Evidence of heterogeneity was considered as justification for using random-effects over fixed-effects models, and the threshold for meeting statistically significant heterogeneity was set at I^2^ > 50% and p < 0.05. Forest plots were generated using the STATA Meta-analysis workflow and Metaprop command (version 16, StataCorp LLC, College Station, TX, USA) [44, 45]. Results from individual studies were weighted according to their standard errors. Comparisons of ACPRs between AL and non-AL treatment arms were performed using sub-group meta-analyses considering the AL and non-AL arms as comparison groups and testing hypotheses for heterogeneity. The type I error threshold for all hypothesis tests was set at 5%.

### Quality assessment

Publication bias was assessed for selected studies using the Cochrane Risk-of-Bias tool [46]. Bias was classified according to randomization processes, deviations from intended interventions, missing outcome data, outcome measurements, and selection of the primary outcome. Risk of bias was classified as ‘low’, ‘unclear’, and ‘high’. Bias assessments were conducted independently by two assessors, and the results were graphed according to the percentage of low concern.

## Results

**Fig. 1:**
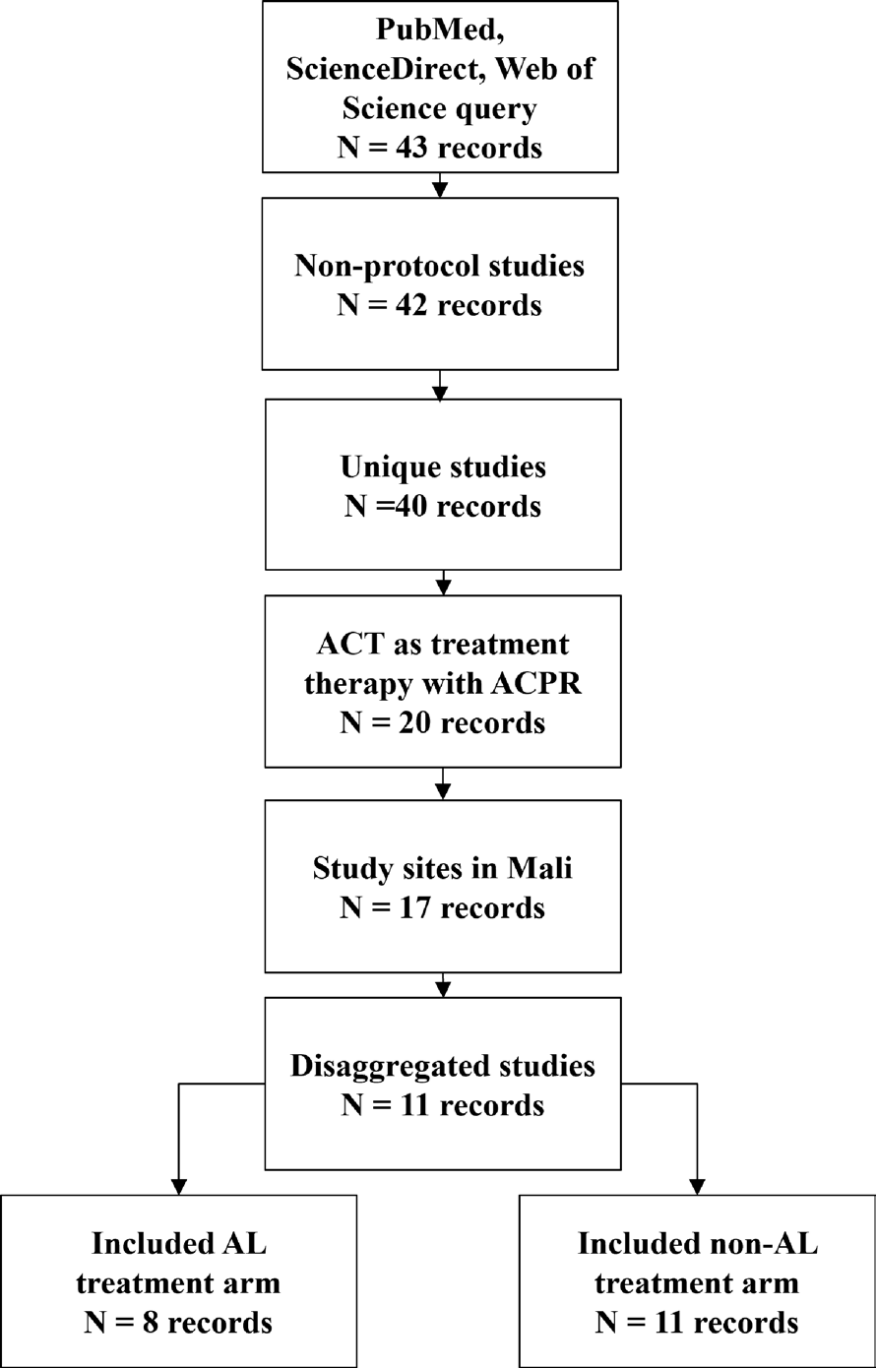
Selection process for included studies. A total of 11 ACT studies were selected for inclusion, where 8 of these studies included an AL treatment arm. ACT: artemisinin-based combination therapy; ACPR: adequate clinical and parasite response; AL: artemether–lumefantrine

A total of 43 publications were identified from bibliographic databases using the PubMed, ScienceDirect and Web of Science search engines. Among these publications, 32 were excluded for the following reasons: protocol studies (one study); replicate results from other selected studies (two studies); ACT was studied as a preventive treatment therapy, the study did not include an ACT arm or did not include reported ACPRcs (20 studies); study did not include Mali study sites (three studies); and, Mali sites could not be de-aggregated from multi-country studies or included replicate results from previously selected studies (six studies). A total of 11 studies met the inclusion criteria [47–59] (Fig. 1). ACPRcs at day 28 was considered in this review as the primary outcome. Characteristics for the 11 selected studies are listed in Table 1.

**Table 1:** Characteristics of selected artemisinin-based combination therapy trials in Mali

No	Author, month, year, ref	Mali Study site	Lat °N	Lon °W	Study characteristics^b^	Drug type	N	n	ACPR (%)	ACPRc (%)
1	Sagara Oct 2006 [47]	Sotuba	12.66	7.91	≥ 6 months; Sep 2003–Jan 2004	AL	303	297	89.6	99.0
						AS + SMP	303	296	98.7	100.0
2	Djimde Mar 2008 [48]	Bougoula-Hameau	10.78	6.92	≥ 5 kg; Dec 2002–Oct. 2004	AS + AQ	252	235	81.3	99.1
						AS + SP	250	232	95.7	100.0
						AS	251	234	57.7	96.5
3	Sagara Nov 2008 [49]	Kambila	12.79	8.11	≥ 10 kg, ≥ 1 yr; Aug 2004–Feb 2005	AL	235	232	67.8	96.9
						AS + MQ	235	232	79.7	96.0
4	Kayentao Jan 2009 [57]	Faladje	13.13	8.33	6–59 months; July 2005–Jan 2006	AS + AQ	133	131	55.7	95.4
						AS + SP	132	130	90.8	96.9
						SP + AQ	132	130	97.7	99.2
5	Sagara Apr 2009 [50]	Bancoumana	12.21	8.26	≥ 6 months; Aug 2006–May 2007	AL	253	85	NR	100.0
		Kolle	12.23	8.24		AS + SMP, 1 day		82	NR	100.0
		Samako	12.26	8.28		AS + SMP, 3 days		86	NR	100.0
6^a^	Ndiaye Jun 2009 [51]	Bancoumana	12.21	8.26	≥ 10 kg; Mar 2006–Dec 2006	AL	67	65	NR	94.0
						AS + AQ, 1 day	66	64	NR	93.9
						AS + AQ, 2 days	68	65	NR	95.6
7	Sagara Jul 2012 [52]	Bougoula-Hameau	10.78	6.92	≥ 6 months; July 2005–July 2007	AL	260	252	62.0	94.4
						AS + AQ	260	252	78.5	95.8
						AS + SP	260	253	89.1	97.9
8	Kayentao Oct 2012 [58]	Bougoula	11.15	7.49	≥ 5 to < 25 kg, ≤ 12 yrs; Nov 2007–Sep 2008	AL	NR	43	NR	100.0
						AS + Pyr	NR	84	NR	100.0
9	Maiga Feb 2015 [59]	Kolle	12.23	8.24	6–59 months; Dec 2004–Dec 2005	AS + SP	306	296	94.9	99.0
		Bancoumana	12.21	8.26		SP + AQ	304	293	98.6	100.0
						SP	302	294	93.5	97.2
10	Niaré Mar 2016 [54]	Banambani	12.79	8.04	≥ 6 months; Oct 2010–Jan 2014	AL	237	228	83.8	98.2
		Sotuba	12.66	7.91		AS + SP	242	228	91.2	100.0
		Kolle	12.23	8.24						
11	Dama Oct 2018 [56]	Doneguebougou	12.81	7.98	≥ 6 months; Nov 2013–Dec 2015	AL	158	155	84.5	98.1
		Torodo	13.06	8.21		DHA + PQ	159	155	97.4	99.4

Column 1 is a sequential number according to the ascending order of month and year of publication. Column 2 shows the last name of the first author and month and year of publication for each study, respectively. Columns 4 and 5 include GPS coordinates (latitude and longitude, respectively). Age and weight range inclusion criteria and study time period are shown in Column 6. Treatment arm, number of subjects enrolled per study arm, and number of subjects evaluated (number of enrolled subjects excluding withdrawals) by study arm are shown in Columns 7, 8 and 9, respectively. ACPRs and ACPRcs (as percentages of non-treatment failures) are listed in Columns 10 and 11. All 11 studies employed supervised drug administration

All results based on per protocol analyses, save for study number 6 (Ndiaye et al. [51]) as country-specific results were only available for intention-to-treat analyses

ACT: artemisinin-based combination therapy; lat.: latitude; lon.: longitude; ACPR: adequate clinical and parasite response at 28 days; ACPRc: polymerase chain reaction-corrected adequate clinical and parasite response at 28 days; CI: confidence interval; NR: Not reported; PCR: polymerase chain reaction; +: joint combination of treatment regimens; Drug types: AL: artemether–lumefantrine; AP: artesunate–pyronaridine; AQ: amodiaquine; AS: artesunate; AS + MQ: artesunate–mefloquine; CD: chlorproguanil–dapsone; DHA: dihydroartemisinin; PQ: piperaquine; Pyr: pyronaridine; SMP: sulfamethoxypyrazine–pyrimethamine; SP: sulfadoxine–pyrimethamine

^a^ Results based on intention-to-treat analyses

^b^ Study characteristics include and weight inclusion criteria and study time period for those studies where this information was available

**Fig. 2:**
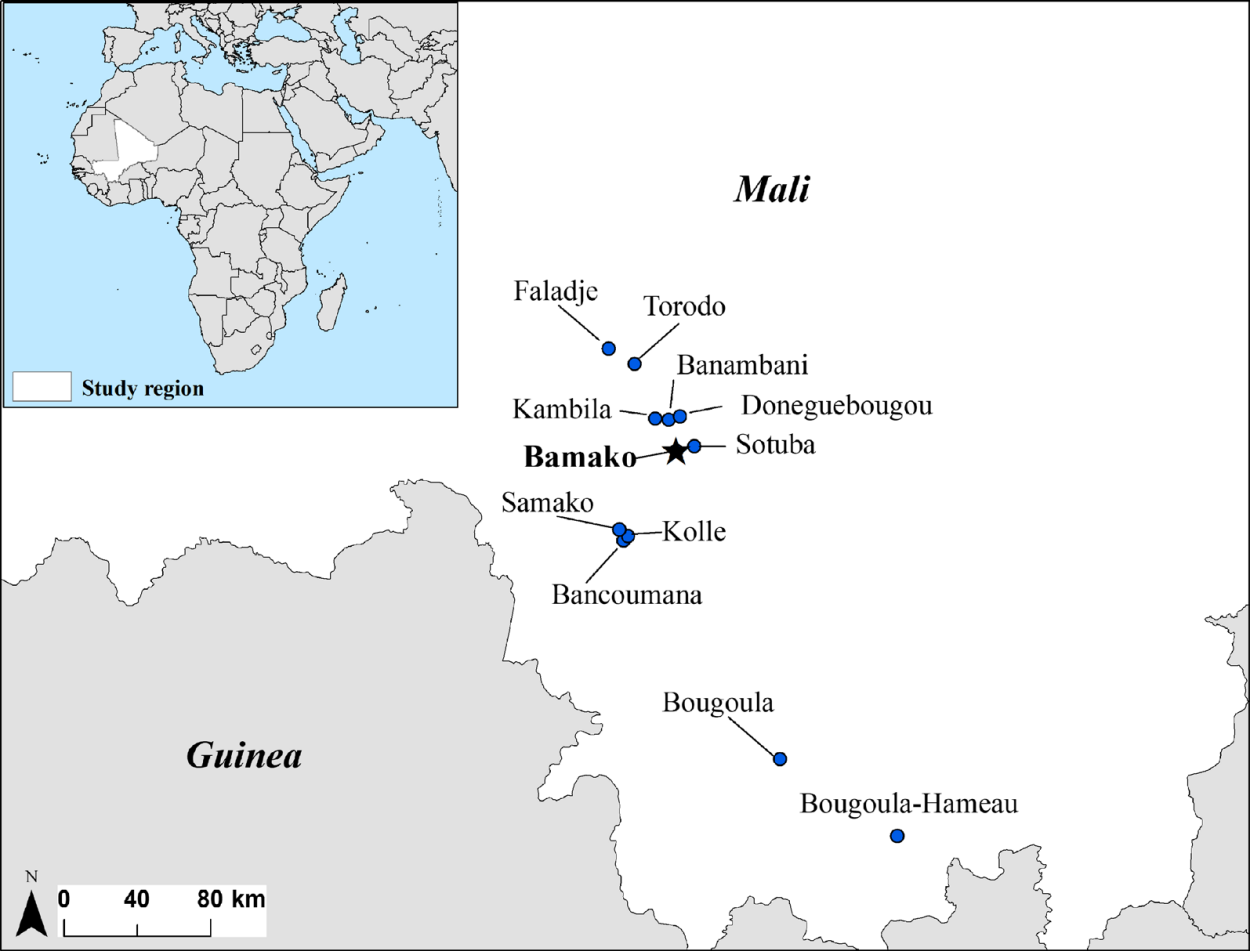
Field study locations for included artemisinin combination therapy trials in Mali. The majority of the study locations were situated in the southern, rural parts of Mali where most of its population resides. ACT: artemisinin-based combination therapy

The 11 selected studies included a total of 28 study arms, including 26 ACT arms and two NACT arms. Ten of the studies included an AL arm, and both of the two NACT arms were partial ACT (non-combinations AS and SP). The total number of subjects evaluated in the selected studies was 5,578, with 1,357 subjects in AL arms and 4,221 subjects in non-AL arms. For the non-AL arms, the number of evaluated subjects were: AS + SP (n = 1,139); AS + AQ (n = 747); AS + Pyr (n = 533); AS + SMP (n = 464); SP + AQ (n = 423); SP (n = 294); AS (n = 234); AS + MQ (n = 232); and, DHA-PQ (n = 155). All of the results were based on per protocol analyses, save for the study by Ndiaye et al. [51] as results were only available for intention-to-treat analyses. The majority of the field study sites for the selected studies were situated in rural or semi-rural southern parts of Mali, where most of its population resides. The locations of the field study sites are shown in Fig. 2.

**Fig. 3:**
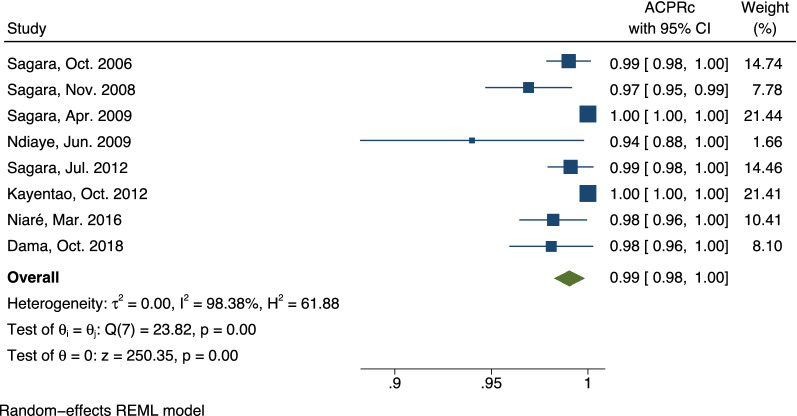
Forest plot of artemether–lumefantrine polymerase chain reaction-corrected adequate clinical and parasite responses for included studies. The pooled ACPRc for AL treatment arms was 99.0% (95% CI 98.3%, 99.8%). ACPRc: polymerase chain reaction-corrected adequate clinical and parasite response for falciparum malaria at 28 days; AL: artemether–lumefantrine; PCR: polymerase chain reaction

Because AL was the first-line ACT in Mali and thus was the most common treatment arm in the selected trials, a stratified meta-analysis was performed for the AL arm (Fig. 3).

**Fig. 4:**
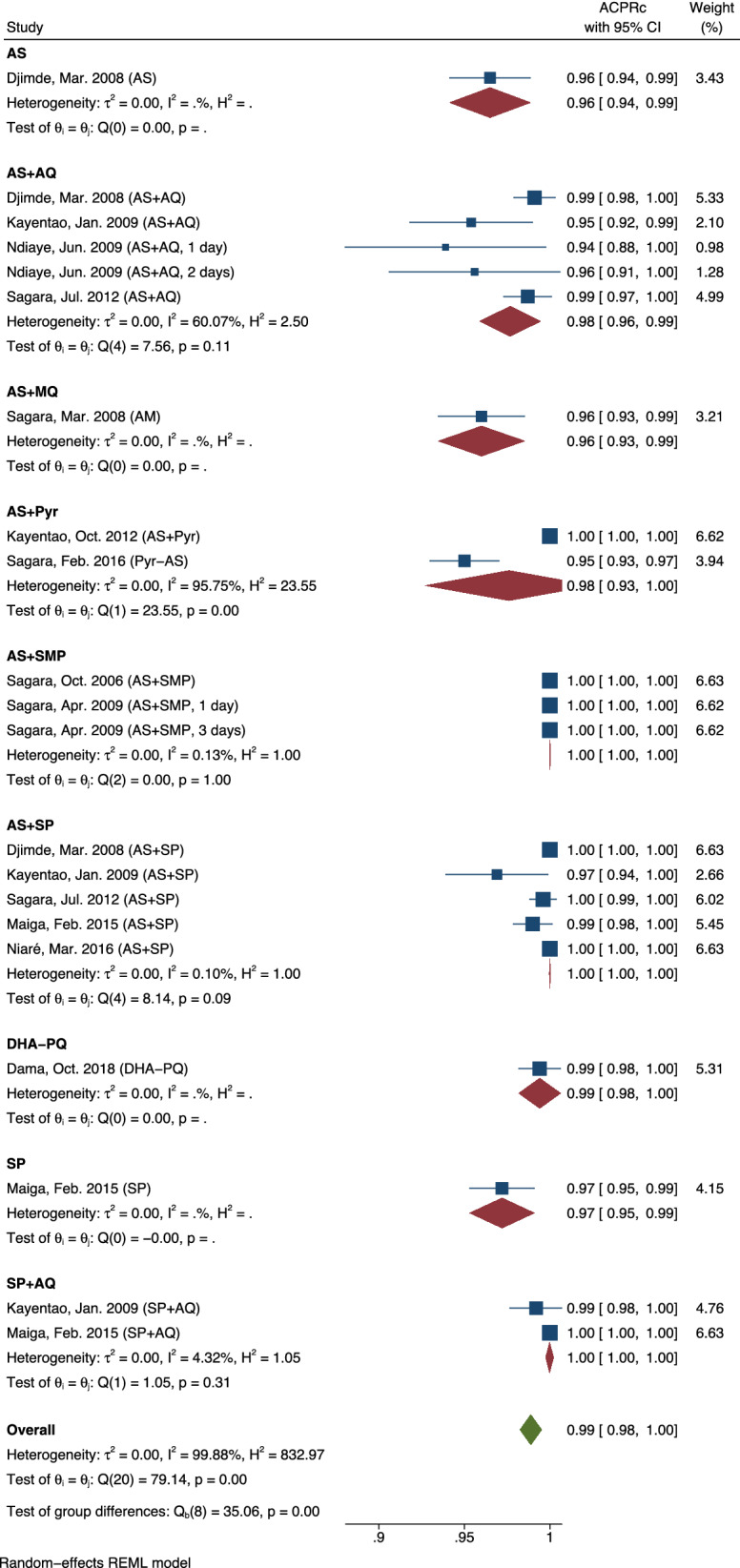
Forest plot of polymerase chain reaction-corrected adequate clinical and parasite responses for studies with non- artemether–lumefantrine treatment arms. Among non-AL treatment arms including at least two studies, AS + AQ and AS + Pyr were the only treatment arms with pooled ACPRcs less than 100.0% (97.7% (95% CI 95.9%, 99.4%) and 97.6% (95% CI 92.7%, 100.0%), respectively. The overall ACPRc for non-AL treatment arms was 98.9% (95% CI 98.3%, 99.5%). Only treatment arms with at least two representative studies are shown. ACPRc: polymerase chain reaction-corrected adequate clinical and parasite response for falciparum malaria at 28 days; AL: artemether–lumefantrine; PCR: polymerase chain reaction

Eight studies were included in the stratified analysis for the AL arm (the other three selected studies did not include an AL arm). The ACPRs ranged from 94 (Ndiaye et al. [51]) to 100% (Kayentao et al. [58]). The hypothesis test for heterogeneity was significant (p = 0.001), and therefore random-effects meta-analyses approaches were used to assess the AL arm. The overall ACPRc for AL was 99.0% (95% CI 98.3%, 99.8%). An analogue, stratified meta-analysis was carried out for non-AL treatment arms (Fig. 4).

The hypothesis test for heterogeneity was also significant for the non-AL meta-analyses (p < 0.001), and therefore random-effects meta-analyses approaches were applied to analyse the ACPRcs for the non-AL arms. The pooled ACPRc for non-AL treatment arms was 98.9% (95% CI (98.3%, 99.5%)). Only one study included a lower 95% confidence bound of less than 95% (Ndiaye et al. [51]; 95% CI (88.0%, 100.0%)), which is potentially attributable to its intent-to-treat analyses. ACPRs for all three studies in the AS + SMP arm were 100% with no reported treatment failures.

### Comparison of AL and non-AL treatment arms

**Fig. 5:**
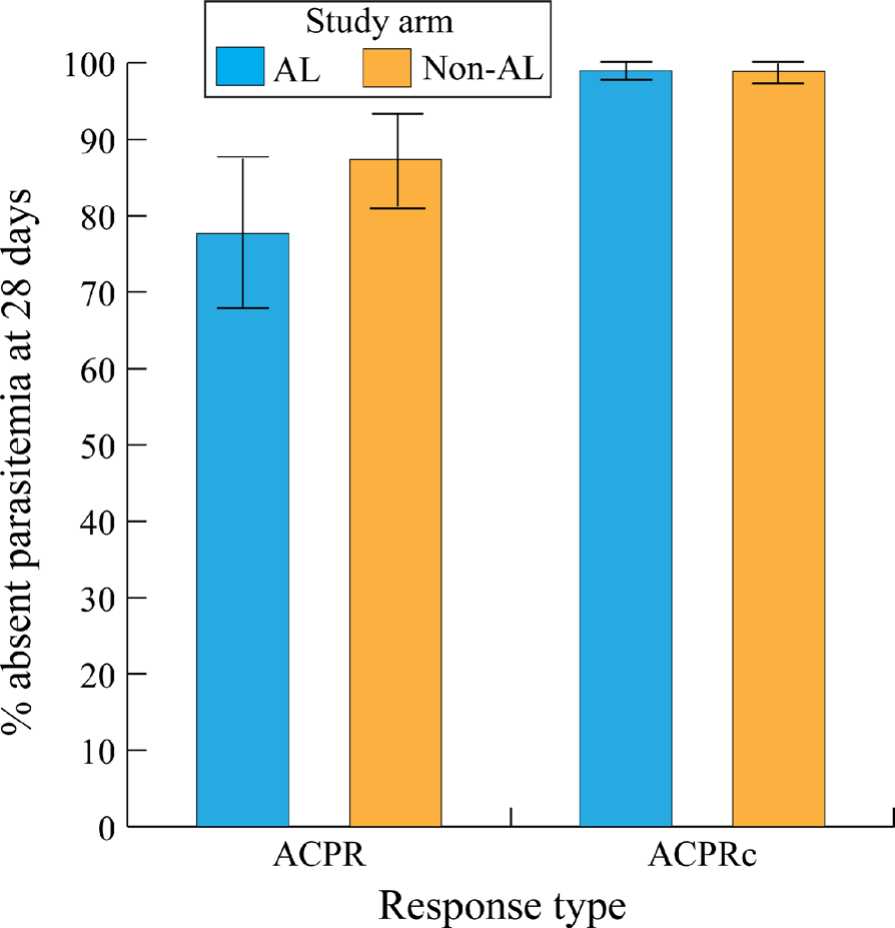
Uncorrected versus corrected malaria polymerase chain reaction-corrected adequate clinical and parasite responses for artemisinin-based combination therapy arms in Mali trials. AL and non-AL treatment arms did not significantly differ according to ACPRs or ACPRcs (p = .120 and p = .752, respectively). The error bars denote the 95% confidence intervals for each combination of treatment arm (AL or non-AL) and outcome (ACPR or ACPRc). ACPR: adequate clinical and parasite response; ACPRc: polymerase chain reaction-corrected adequate clinical and parasite response for falciparum malaria at 28 days; ACT: artemisinin-based combination therapy

Figure 5 shows treatment success rates according to ACPR and ACPRc 28 days following treatment by treatment type (classified as AL and non-AL arms).

The overall ACPRs and 95% confidence intervals for the AL and non-AL groups were 77.7% (67.2%, 88.1%) and 87.4% (81.0%, 93.8%), respectively. Sub-group meta-analysis tests of heterogeneity revealed that the AL and non-AL treatment arms were not statistically different with respect to ACPRc or ACPR outcomes (p = 0.752 and p = 0.120, respectively). However, the ACPRs were significantly lower than the ACPRcs for both the AL and non-AL treatment arms (p < 0.001 and p < 0.001, respectively).

### Publication bias assessment

**Fig. 6:**
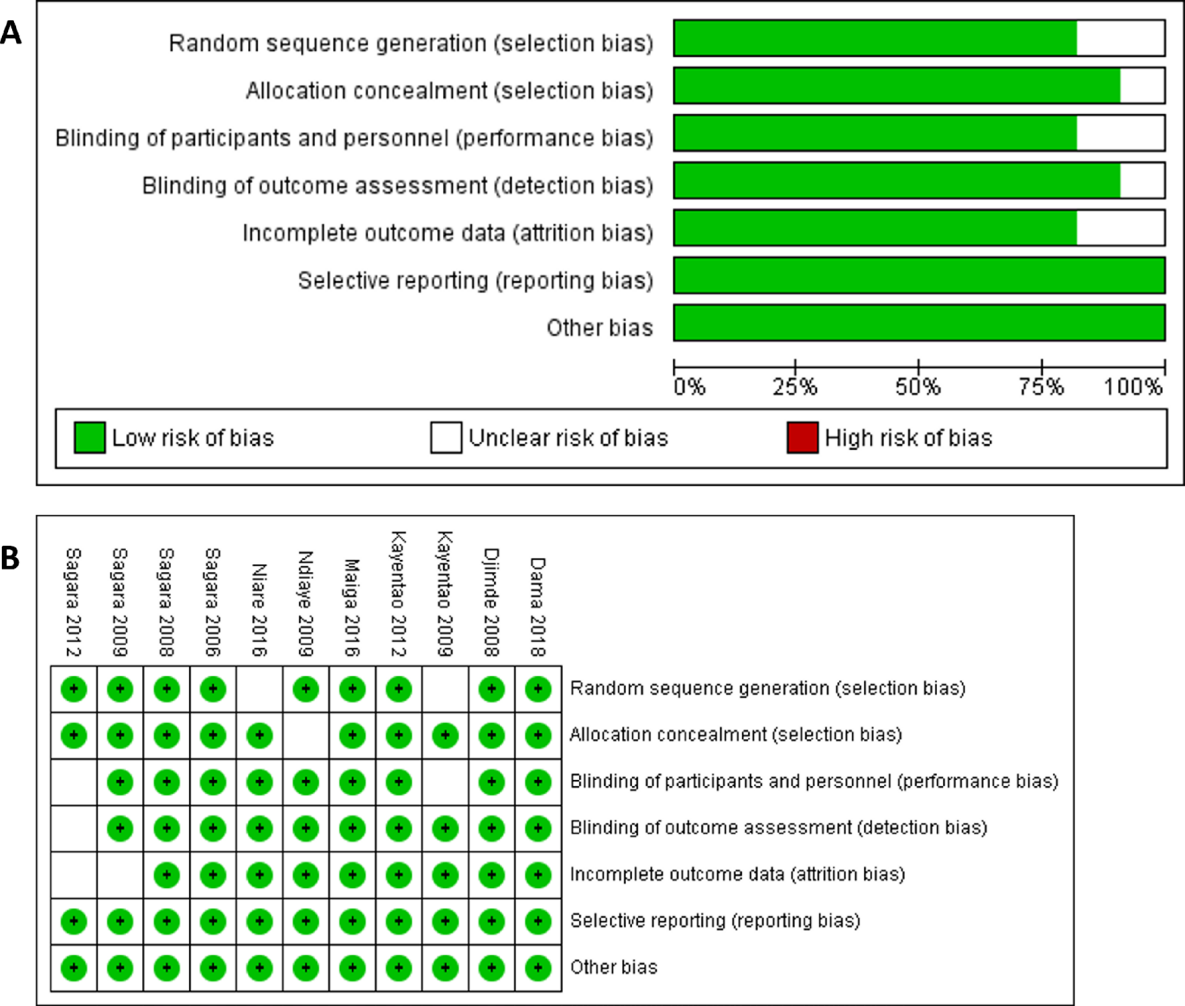
Risk of bias assessment for included studies. **A** Shows the risk of bias graph, and **B** shows the Risk of bias summary figure. High risk of bias was not reported for any of the criteria in any of the studies, and 63.6% (7/11) of the studies reported low risk of bias for all assessed criteria

Bias assessments were conducted independently by two of the co-authors for this review paper, and six major assessment criteria were evaluated: selection bias (random sequence generation and allocation concealment); performance bias (blinding of participants and personnel); detection bias (blinding of outcome assessment; attrition bias (incomplete outcome data); and, reporting bias (selective reporting; and a general category for other types of bias. The publication bias assessment results were graphed as the proportion of reported low risk of bias according to the guidelines in the Cochrane Review Manager application (Fig. 6).

The majority of the included studies showed high quality. Six out of the 11 studies (54.5%) were scored as having no bias according to the six criteria. For those cases where bias was reported, it was most commonly observed for the random sequence generation and blinding of participants' criteria (each of these criteria included two out of the 11 studies (18.2%) with an unclear risk of bias). No selective reporting bias or high risk of bias was observed in any of the studies.

## Discussion

ACT remains highly efficacious in treating uncomplicated falciparum malaria in Mali. The extremely high efficacy in both AL and non-AL treatment arms reported here suggests that non-AL therapy lines are available as potential alternatives to AL in the event of drug resistance or supply shortage. While the AL and non-AL treatment arms did not significantly differ according to ACPRcs 28 days following treatment, the lack of difference may suggest that non-AL treatment arms are at least equally efficacious as AL in Mali for treating uncomplicated falciparum malaria. Artemisinin resistance has recently been observed in Southeast Asia [60] and on at least one occasion in Africa [34], and recent studies for Rwanda have shown partial artemisinin resistance [37]. Together, these findings of potential artemisinin resistance suggest that country-specific review studies will play a key role in monitoring anti-malarial drug resistance patterns. Additionally, country-specific review studies on ACT are needed to complement more extensive multi-country meta-analyses to evaluate potential aggregation bias introduced in multi-country studies. Carrying out this strategy, however, is contingent upon regularly carrying out clinical trials or high-quality observational studies that would ideally adequately represent both rural and urban locations. It is worth adding that several available treatment arms were either not previously tested at Mali locations or were not available based on the selection criteria, including AS + CD, and artesunate–pyronaridine (AP). AS + SMP was last evaluated in Mali approximately 11 years ago [2009], so the results here may not reflect its current efficacy.

### Reported artemisinin resistance calls for increased evaluation of ACT

Companion studies on alleles known to affect anti-malarial drug resistance patterns provide additional context for the results presented in this work. Regular monitoring of alleles such as *kelch13* has been shown to be the key reasons for artemisinin resistance in Southeast Asia, and more recently, in Uganda and are needed to be assessed through field studies such as those by Diakité et al. [61]. While drug resistance has been reported in Southeast Asia, such resistance has not been reported in Mali [61], which is consistent with the high efficacy rates reported here. However, the recent reports in Rwanda regarding partial artemisinin resistance may suggest the emergence of artemisinin resistance in Africa [40]. It is worth mentioning that the introduction of ACT may, in turn, lead to reduced resistance in earlier first-line regimens, such as chloroquine, due to their decreased usage.

Also worth mentioning is that parasitological responses shortly following treatment are important measures for early detection of resistance. The early manifestation of resistance may become evident from slow parasitological responses according to measures such as parasite clearance half-lives. While data on early parasitological response were unavailable here, such data would have great utility for fully assessing drug resistance patterns. The assessment of early parasitological responses is perhaps most useful when efficacy rates are high, as was observed here for Mali study locations before resistance becomes widespread.

### Implementation of ACT differs outside of controlled settings

The studies analysed here were performed under controlled experimental settings and were based on per protocol analyses. The results focused on ACT efficacy in terms of ACPRcs and did not consider potential confounding factors such as treatment compliance, side effects, drug cost, and drug quality. Also, malaria is often an assumed illness for symptomatic febrile subjects in malaria-endemic countries, and symptomatic subjects commonly receive anti-malarial treatment therapy for unconfirmed malaria or unknown febrile illnesses in the absence of diagnostic testing. Delivery approaches (fixed-point delivery at public health units or door-to-door delivery) may also directly impact patient compliance to drug instructions and recommended usage. For these reasons, the ability to implement anti-malarial treatment strategies in practical terms should be considered along with their performance under controlled settings. These types of limitations have recently been noted for Burkina Faso as limiting factors for investigating current artemisinin resistance [35].

### Non-artemisinin-based combination therapy such as aminoquinoline-13 may provide plausible alternative therapy

Aminoquinoline-13, or AQ-13 is an analogue of chloroquine that is active against chloroquine-resistant *Plasmodium* species [62]. Phase II trials have shown the regimen to be non-inferior to the AL ACT according to per-protocol analyses with no treatment failures at 28 days following treatment [55]. The regimen is gaining considerable support for phase III clinical trials, specifically as a candidate treatment of uncomplicated falciparum malaria as a partner drug in combination therapy [63].

### Utility of pharmacy data sources in monitoring ACT efficacy

Pharmacy consultations and inventories offer potentially valuable data sources for monitoring ACT efficacy spatial and temporal patterns. Pharmacies maintain a wealth of information on ACT and are often the first point of contact for febrile subjects. However, pharmacy data sources are rarely adequately monitored or considered as a means for measuring community health. The cost reduction of smartphones presents opportunities for building automated systems for capturing pharmacy data to monitor ACT and improve associated adherence and compliance. Data platforms are needed to capture data within the pharmacies and link them with their associated public health units. Through such platforms, pharmacy supply and clinical presentation data sources provide a potential approach for carrying out syndromic surveillance activities through surveillance of pharmacy presentations and monitoring drug supplies and inventories.

### Study strengths and potential limitations

A particular strength of this work is its direct focus on Mali study sites. This work was made possible through the numerous field studies carried out in Mali over the past several decades. However, the study had several limitations. First, the selection approach for this study included only published works maintained in bibliographic databases available through the PubMed, ScienceDirect and Web of Science search engines, which did not capture unpublished studies or studies indexed through those bibliographic databases. Also, the studies contributing to this review paper were published between 2006 and 2018, and lag periods between the time of the field study to publication usually exceeded 1 year. Several studies contributed multiple comparison arms, and intra-trial dependence was not accounted for in the meta-analyses. Finally, several of the studies here occurred prior to the 2009 WHO protocol guidelines [64] for determining ACT efficacy that was applied in the studies following the establishment of these guidelines.

## Conclusions

ACT remains highly efficacious in Mali, and the results here suggest that AL will continue to be a viable treatment option in Mali for the foreseeable future. These findings also suggest that potential ACT alternatives may be at least equally efficacious as first-line AL therapy. Country-specific meta-analyses on ACT play a central role in monitoring and evaluating drug efficacy patterns and for guiding local malaria treatment policies, particularly in the wake of reported partial artemisinin resistance in Rwanda.

## Data Availability

All data generated or analyzed during this study are included in this article and its supplemental materials.

## Abbreviations

ACE: African Centres of Excellence in Bioinformatics
ACPR: Adequate clinical and parasite response
ACT: Artemisinin-based combination therapy
AL: Artemether–lumefantrine
AP: Artesunate–pyronaridine
AQ: Amodiaquine
AQ-13: Aminoquinoline-13
AS: Artesunate
AS + MQ: Artesunate–mefloquine
CD: Chlorproguanil–dapsone
CQ: Chloroquine
DHA: Dihydroartemisinin
GPS: Global positioning system
IPT: Intermittent preventive treatment
ITT: Intention to treat
NACT: Non-artemisinin-based combination therapy
P.: Plasmodium
PCR: Polymerase chain reaction
PP: Per protocol
PQ: Piperaquine
Pyr: Pyronaridine
RCT: Randomized controlled trial
SE: Standard error
SMP: Sulfamethoxypyrazine–pyrimethamine
SP: Sulfadoxine–pyrimethamine
USTTB: University of Sciences, Techniques and Technologies of Bamako, Mali
WHO: World Health Organization
